# Montelukast, an Anti-asthmatic Drug, Inhibits Zika Virus Infection by Disrupting Viral Integrity

**DOI:** 10.3389/fmicb.2019.03079

**Published:** 2020-01-30

**Authors:** Yongkang Chen, Yuan Li, Xiaohuan Wang, Peng Zou

**Affiliations:** Shanghai Public Health Clinical Center, Fudan University, Shanghai, China

**Keywords:** Zika virus, flavivirus, montelukast, viral inactivator, viral integrity

## Abstract

The association of Zika virus (ZIKV) infection and severe complications including neurological sequelae especially fetal microcephaly has aroused global attentions since its outbreak in 2015. Currently, there are no vaccines or therapeutic drugs clinically approved for treatments of ZIKV infection, however. And the drugs used for treating ZIKV in pregnant women require a higher safety profile. Here, we identified an anti-asthmatic drug, montelukast, which is of safety profile for pregnant women and exhibited antiviral efficacy against ZIKV infection *in vitro* and *in vivo*. And we showed that montelukast could disrupt the integrity of the virions to release the viral genomic RNA, hence irreversibly inhibiting viral infectivity. In consideration of the neuro-protective activity that montelukast possessed, which was previously reported, it is promising that montelukast could be used for patients with ZIKV infection, particularly for pregnant women.

## Introduction

Since the first isolation in 1947 from the rhesus macaque in Zika forest, Uganda, and publication of the first human case of infection in 1952, Zika virus (ZIKV), a mosquito-borne enveloped RNA virus, has become a member of *Flavivirus* genus in the Flaviviridae family for more than 70 years together with dengue virus (DENV), yellow fever virus (YFV), Japanese encephalitis virus (JEV), and West Nile virus (WNV). The infection of ZIKV has aroused global awareness only in recent years because of some severe neurological complications such as Guillain–Barré syndrome (Brasil et al., 2016; Peixoto et al., 2019) and congenital Zika syndrome (Baud et al., 2017; Gurung et al., 2019), which consists of many clinical manifestations including intracranial calcification (ICC) and cerebellar hypoplasia (de Fatima Vasco Aragao et al., 2016; Oliveira Melo et al., 2016; Cui et al., 2017), and is remarkably typified by microcephaly found both in humans and in animal models (Cui et al., 2017), although symptoms of majority of infection with ZIKV are mild or asymptomatic. In addition, infection with ZIKV leads to impaired human spermatozoa production demonstrated by decreased sperm count in the early stage of ZIKV infection (Joguet et al., 2017). And even 1 year after ZIKV infection, abnormal spermogram results could still be observed (Avelino-Silva et al., 2018).

Since the local outbreak in Brazil and quick spread to other countries in 2015, the effort for seeking inhibitors suitable for treatment of ZIKV is ongoing till now. Targeting different stage of ZIKV life cycle, some inhibitors have been discovered (Wang et al., 2017). The first step of ZIKV infection is its attachment to the host cell membrane. A peptide derived from the stem region of E protein of ZIKV blocked the binding of virions to cells via its interaction with E proteins to disrupt the integrity of the viral membrane (Yu et al., 2017). Erythromycin estolate was also found to effectively inhibit ZIKV infection by disrupting the integrity of the viral membrane (Wang et al., 2019). ZINC33683341 and curcumin also inhibit infection of ZIKV by disturbing the interaction between virions and cells (Delvecchio et al., 2016; Fernando et al., 2016; Li et al., 2017b; Mounce et al., 2017). Nanchangmycin, one of the antibiotics against gram-positive bacteria, inhibited ZIKV infection through blocking clathrin-mediated endocytosis (Rausch et al., 2017). A natural oxysterol, 25-hydroxycholesterol also inhibited ZIKV infection probably owing to the obstruction of the membrane fusion mediated by E protein (Li et al., 2017a). Some inhibitors can interfere with the viral RNA replication to break off the viral life cycle. For example, 7-deaza-2′-*C*-acetylene-adenosine (NITD008), an adenosine analog, inhibited ZIKV and DENV replications in a dose-dependent manner *in vitro* by terminating viral RNA synthesis and protected mice from ZIKV infection (Yin et al., 2009; Deng et al., 2016a). Emricasan, a pan-caspase inhibitor, held back the increase in caspase-3 activity induced by ZIKV infection and protected neural progenitors (Xu et al., 2016). By screening about 100 Food and Drug Administration (FDA)-approved pregnancy category B drugs, we identified montelukast, an anti-asthmatic drug. The antiviral activities of montelukast against ZIKV and other two flaviviruses, DENV and YFV, *in vitro* were evaluated. Montelukast also exhibited protective efficacy against ZIKV vertical transmission and lethal challenge. The underlying mechanisms for infectivity inhibition of flaviviruses caused by montelukast were investigated as well in this study.

## Materials and Methods

### Cells, Viruses, and Compounds

BHK-21 cells (Baby Hamster Kidney cells), Vero E6 cells (African green monkey kidney cells), RD cells (rhabdomyosarcoma cells), and human astrocytoma cell line U-251 MG were cultured in Dulbecco’s modified Eagle’s medium (DMEM; Biological Industries, Israel) supplemented with 10% fetal bovine serum (FBS; Biological Industries, Israel) at 37°C and 5% CO_2_. The C6/36 mosquito cells were grown in DMEM containing 10% FBS at 28°C with 5% CO_2_.

ZIKV strain SZ01/2016 (GenBank number: KU866423), which was isolated from a patient who returned from Samoa and was kindly provided by Dr. Cheng-Feng Qin (Deng et al., 2016b); ZIKV strains FLR [#VR1844, American Type Culture Collection (ATCC)] and MR766 (#VR1838, ATCC) obtained from ATCC; DENV-2 kindly provided by Drs. Yunwen Hu and Zhigang Song at the Shanghai Public Health Clinical Center; and YFV strain 17D obtained from Beijing Tiantan Biological Products, Ltd., were all propagated in C6/36 cells as described previously (Yu et al., 2017). The replication-competent vesicular stomatitis virus (VSV) containing an additional viral transcriptional unit coding green fluorescent protein (VSV-GFP) was kindly provided by Dr. Nannan Wu (Wu et al., 2019) and propagated in Vero E6 cells. The enterovirus 71 (EV71) was kindly provided by Dr. Shuye Zhang (Yuan et al., 2018) and propagated in RD cells.

Montelukast sodium and chloroquine phosphate were purchased from Sigma-Aldrich (St. Louis, MO, United States). 7-Deaza-2′-*C*-acetylene-adenosine (NITD008) and curcumin were purchased from MedChemExpress (Monmouth Junction, NJ, United States). Montelukast (Mon), NITD008, and curcumin were dissolved in dimethyl sulfoxide (DMSO). Chloroquine phosphate was dissolved in sterilized water, and all of the dissolved compounds were stored at −20°C.

### Plaque Assay

Plaque assay was performed on BHK-21 cells or Vero E6 cells as previously described (Yu et al., 2017). Briefly, BHK-21 or Vero E6 cells were seeded onto cell culture plates and incubated overnight to a confluent monolayer. Virus or a mixture of virus and compounds was added into the wells and then incubated for 2 h. The supernatant was then removed, and the wells were washed and covered with an overlay of DMEM containing 0.6% low-melting-point agarose (LMP agarose, Promega, United States) and 2% FBS. The plates were then further incubated for approximately 5 days until the plaque developed. For VSV-GFP, the plates were incubated for 1 day for plaque development. After being fixed with 4% formaldehyde and stained with 1% crystal violet, the plaques were visualized, and the plaque-forming units (pfu) were counted.

### Assay for Antiviral Activity

BHK-21 cells or Vero E6 cells were seeded in six-well plates and allowed to adhere overnight. Montelukast sodium serially diluted in serum-free DMEM and 50 pfu of viruses were mixed and incubated 1 h at room temperature before being added to each well of cells. After incubation at 37°C for 2 h, the plaque assay was performed, and the plaques were visualized as described above. Curcumin, a reported antiviral drug (Mounce et al., 2017), was included as positive control. The percent inhibition by the montelukast was calculated, and the 50% inhibitory concentration (IC50) value was determined by using the software CalcuSyn (Chou and Talalay, 1984).

### Flow Cytometry Experiments

U-251 MG cells were infected with ZIKV strain SZ01 at a multiplicity of infection (MOI) of 1 after viruses incubating with serially diluted montelukast for 1 h. Then, the inoculum was removed 2 h later, and fresh DMEM containing 2% FBS and serially diluted montelukast was supplemented. Cells were trypsinized, fixed, and permeabilized with BD Fixation/Permeabilization Kit (BD Biosciences, United States) at 40 h post-infection (hpi) and then stained with anti-E mAb 4G2 (10 μg/ml); and a rabbit anti-mouse IgG, which was coupled to fluorescein isothiocyanate (FITC) (DAKO, Denmark) and diluted 1:400. Flow cytometry experiments were carried out in an LSRFortessa cell analyzer (BD Biosciences), and samples were analyzed using FlowJo software version 10 (TreeStar).

### Cytotoxicity Assay

BHK-21, Vero E6, and U-251 MG cells grown in 96-well plates (2 × 10^4^ cells/well) were treated with serially diluted montelukast in DMEM containing 2% FBS for 48 h at 37°C. Cell Counting Kit-8 (CCK-8; Dojindo, Japan), a water-soluble non-radioactive reagent, allowing sensitive colorimetric assays for the determination of cell viability, was used to evaluate cytotoxicity according to the instruction manual. The absorbance at 450-nm wavelength was measured by the iMark^TM^ microplate reader (Bio-Rad, United States). The percent cytotoxicity was calculated, and the 50% cytotoxicity concentration (CC50) value was determined by the CalcuSyn (Chou and Talalay, 1984).

### Time of Addition Experiments

To determine at which stage the montelukast displayed inhibitory efficiency, the time of addition assay was performed as previously described (Du et al., 2009; Liu et al., 2015). BHK-21 cells confluent in the six-well cell culture plates were infected with 50 pfu of virus; montelukast (10 μM) was added to the infected cells at 0, 1, 2, 4, and 8 hpi. Then, the supernatant was replaced with DMEM containing 0.6% LMP agarose and 2% FBS at 16 hpi. The plaque assay was performed, and the plaques were visualized and counted as described above.

### Assay for Virus Adsorption

To test whether montelukast inhibited virus attachment, the assay for virus adsorption was performed as previously described (Talarico et al., 2005). BHK-21 cells confluent in six-well plates were infected with 500 pfu of virus in the presence or absence of 10 μM of the montelukast and incubated for 1 h on ice. The curcumin (10 μM), which showed an antiviral activity at the stage of virus adsorption (Mounce et al., 2017), was included as control. Supernatant containing unadsorbed virus was discarded, cells were then washed twice with ice-cold DMEM and DMEM containing 0.6% LMP agarose, and 2% FBS was overlaid. Virus plaques were visualized and counted as described above.

### Assay for Virus Internalization

The virus internalization assay was performed as previously described (Talarico et al., 2005; Si et al., 2018). BHK-21 cells confluent in six-well plates were infected with 500 pfu of viruses on ice. After 1 h of virus adsorption, unadsorbed virus was discarded, and cells were then washed with ice-cold DMEM and transferred to 37°C in the presence or absence of 10 μM of the montelukast. The chloroquine phosphate (50 μM), which showed an antiviral activity at the stage of virus internalization (Delvecchio et al., 2016; Li et al., 2017b), was included as control. After 2 h of incubation, cells were washed with DMEM and covered with DMEM containing 0.6% LMP agarose and 2% FBS. Virus plaques were visualized and counted as described above.

### Assay for Viral RNA Replication

To test whether montelukast inhibited viral RNA replication, an assay was performed at 4 hpi, a time after entry has occurred (Rausch et al., 2017). Briefly, BHK-21 cells confluent in six-well plates were infected with 50 pfu of virus at 37°C. After 4 h of incubation, the supernatant was removed and replaced with DMEM in the presence or absence of 10 μM of the montelukast. The NITD008, which showed an antiviral activity at the stage of virus RNA replication (Deng et al., 2016a), was included as control. After incubation for additional 12 h, supernatant was removed and cells were washed with DMEM and overlaid with DMEM containing 0.6% LMP agarose and 2% FBS. Virus plaques were visualized and counted as described above.

### Infectivity Inhibition Reversibility Assay

To test whether the inhibition of montelukast on flavivirus infection is reversible, the infectivity inhibition reversibility assay was performed as previously described with some modification (Lok et al., 2012). Virus measuring 100 pfu was incubated with 10 μM of montelukast in a total volume of 10 μl of DMEM for 1 h at room temperature. Immediately before being added to BHK-21 cell monolayer, the virus/montelukast mixtures were diluted with DMEM to 1 ml, reducing the concentration of montelukast to 0.1 μM. The virus constantly treated with 10 or 0.1 μM of montelukast was included as control. The plaque assay was performed, and virus plaques were visualized and counted as described above.

### RNase Digestion Assay and Quantitative Reverse Transcription PCR

To detect whether montelukast can release the genomic RNA from the flavivirus particles, the RNase digestion assay and quantitative reverse transcription (qRT) PCR were performed as previously described (Lok et al., 2012; Yu et al., 2017). Briefly, montelukast at different concentration was incubated with ZIKV (300 pfu) at 37°C for 2 h. Then, the RNA released from virus was digested by micrococcal nuclease (New England BioLabs, Rowley, MA, United States) for 1 h at 37°C. Then, the viral genomic RNA inside the unbroken virus was extracted by using the EasyPure Viral DNA/RNA Kit (Transgen Biotech, Beijing, China) and detected by qRT-PCR using TransScript Green One-Step qRT-PCR SuperMix (Transgen Biotech, Beijing, China) and the CFX96^TM^ Real-Time System (Bio-Rad, United States) in accordance with the manufacturers’ instructions. The virions of ZIKV, DENV-2, and YFV were also purified for RNase digestion assay by polyethylene glycol (PEG) precipitation as described previously (Yu et al., 2017). Briefly, 50% PEG-8000 (Amresco, United States) and 5M of NaCl were added to the virus at final concentration of 10% and 0.67M, respectively. After incubation on ice overnight, the mixture was centrifuged at 20,200 *g* for 1 h. The supernatant was discarded, and the pellet containing the virions was washed with 3% PEG-8000 in PBS containing 10 mg/ml of bovine serum albumin (BSA) (Amresco, United States). After centrifugation, the pellet was resuspended in PBS, and the RNase digestion assay was performed again. The EV71, an unenveloped RNA virus, was included as negative control. The primers used to detect the RNA sequences of ZIKV, DENV-2, YFV, and EV71 were described previously (Lok et al., 2012; Fernandes-Monteiro et al., 2015; Fischer et al., 2017; Yu et al., 2017; Wu et al., 2018; Yuan et al., 2018) and listed in Table 1.

**TABLE 1:** The primers employed to detect the viral RNA in RNase digestion assay.

Virus	Primer name	Primer sequence (5′ → 3′)
ZIKV SZ01	PrM F1	CTTGGACAGAAACGATGCTGGG
	PrM R1	TGATGGCAGGTTCCGTACACAA
	E F1	TGGAGGCTGAGATGGATGG
	E R1	GAACGCTGCGGTACACAAGGA
	Cap F1	TCACGGCAATCAAGCCATCACT
	Cap R1	GCCTCGTCTCTTCTTCTCCTT
DENV-2	5-1F	AATCCCACCAACAGCAGGGATACT
	5-1R	CGCCATCACTGTTGGAATCAGCAT
	5M-1F	AAGCAGAACCTCCATTCGGAGACA
	5M-1R	AAACACTCCTCCCAGGGATCCAAA
	3M-2F	TCACCAAATCCCACGGTAGAAGCA
	3M-2R	AGGGCATGTATGGGTTGAGAACCT
YFV 17D	F3	GGCAATAAACACATTTGGATTAAT
	R3	CATATTGACGCCCAGGGT
	YFVdual-fwd-vac	GGGACTAGCGTGATCATTGA
	YFVdual-rv-vac	GAATAACTTTCCCGCTATCCGT
	F	GCACGGATGTAACAGACTGAAGA
	R	CCAGGCCGAACCTGTCAT
EV71	F	GCAGCCCAAAACAACTTCAC
	R	AATTTCAGCAGCTTGGAGTGC

*ZIKV, Zika virus; DENV, dengue virus; YFV, yellow fever virus.*

### Ethics Statement

All animal experiments were carried out according to ethical guidelines and approved by Shanghai Public Health Clinical Center Laboratory Animal Welfare & Ethics Committee (2016-A021-01).

### Antiviral Efficacy of Montelukast in Pregnant C57BL/6 Mice

Antiviral efficacy of montelukast in pregnant mice was determined as previously described (Wu et al., 2016; Yu et al., 2017). Briefly, 24 pregnant C57BL/6 mice (E12–14) were randomly assigned into two groups and infected intraperitoneally (i.p.) with 1 × 10^5^ pfu of ZIKV (SZ01). One hour later, the infected mice were i.p. administered with montelukast dissolved in PBS at 50 mg/kg of body weight (*n* = 12) or PBS vehicle control (*n* = 12). Mice were bled retro-orbitally to measure viremia by qRT-PCR at day 1 post-infection. Two embryos were collected randomly from each pregnant mouse, and the viral RNA loads in fetal head of each collected embryo and placenta were determined by qRT-PCR.

### Antiviral Efficacy of Montelukast in A129 Mice

To evaluate the antiviral efficacy of montelukast *in vivo*, A129 mice were used as previously described (Yin et al., 2009; Deng et al., 2016a; Shan et al., 2017; Yu et al., 2017; Tai et al., 2019). Briefly, 16 A129 mice of 4-week-old were randomly assigned into two groups and infected i.p. with ZIKV strain SZ01 at a dose of 1 × 10^5^ pfu each mouse. One hour later, the infected A129 mice were then i.p. administered with montelukast dissolved in PBS at 50 mg/kg of body weight (*n* = 8) or PBS vehicle control (*n* = 8). The treatment was performed once a day for six consecutive days, followed by daily observations of mortality of mice. It was deemed to be protected if mouse survived to 21 days post-infection (dpi). The viral RNA loads of sera on 2 dpi were measured by qRT-PCR.

### Statistical Analysis

All statistical analyses were carried out by using GraphPad Prism 7.0 (GraphPad Software, Inc.). Statistical methods were listed as follows: the log-rank (Mantel–Cox) test to evaluate difference in survival; the non-parametric Mann–Whitney test to examine differences of viral RNA loads in the sera, fetal placenta, or fetal head; and Student’s unpaired two-tailed *t*-test in other cases. Significant difference was defined as *P* < 0.05. ^∗^*P* < 0.05; ^∗∗^*P* < 0.01; ^∗∗∗^*P* < 0.001; ^****^P < 0.0001.

## Results

### Montelukast Inhibited Infection of Zika Virus Strains From the Asian and African Lineages, Dengue Virus, and Yellow Fever Virus in Different Host Cells

**FIGURE 1:**
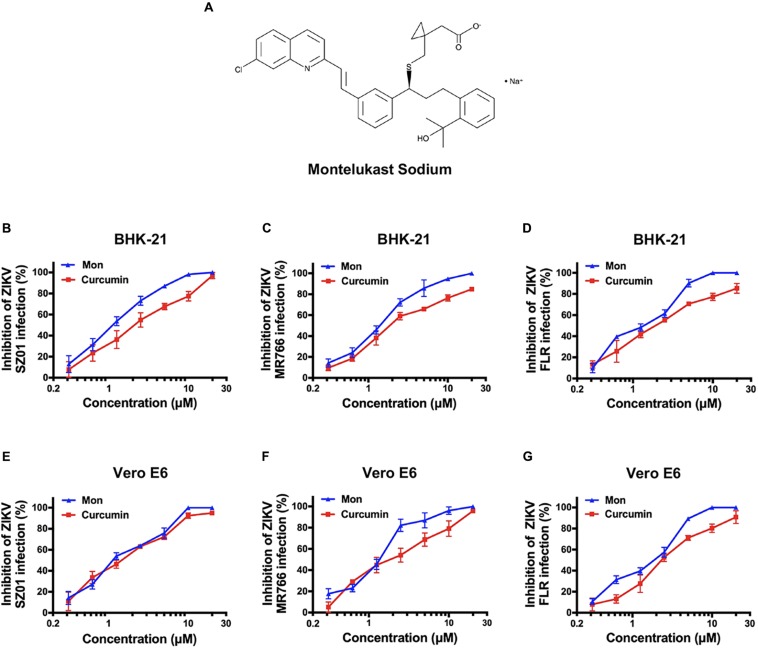
The chemical structure of montelukast and its inhibitory activity against Zika virus (ZIKV) strains from Asian and African lineages in two host cells. **(A)** Chemical structure of montelukast. Dose-dependent inhibition of ZIKV strain SZ01 **(B)**, MR766 **(C)**, and FLR **(D)** infection by montelukast in BHK-21 cells and inhibition of ZIKV strain SZ01 **(E)**, MR766 **(F)**, and FLR **(G)** infection in Vero E6 cells. Curcumin, an anti-ZIKV drug, was included as positive control. All experiments were carried out in triplicate, and the error bars stand for standard deviation (SD). The 50% inhibitory concentration (IC50) is presented as means ± SD and summarized in Table 2.

**FIGURE 2:**
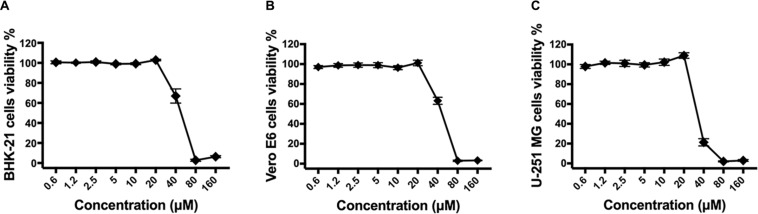
The cytotoxicity of montelukast in different cell lines. BHK-21 **(A)**, Vero E6 **(B)**, or U-251 MG **(C)** cells were treated with serially diluted montelukast. Then cells viability were determined 2 days later by Cell Counting Kit-8 (CCK-8) kit according to the instruction manual. All experiments were carried out in triplicate, and the error bars stand for standard deviation (SD). The 50% cytotoxicity concentration (CC50) is presented as means ± SD and summarized in Table 3.

To identify drugs that could inhibit the infection of ZIKV, an FDA-approved drug repurposing screening was performed. BHK-21 cells were infected with mixture of ZIKV strain SZ01 and drugs at the final concentration of 10 μM. When the ZIKV-induced cytopathic effect (CPE) of BHK-21 cells was obvious, CCK-8, a highly water-soluble non-radioactive reagent like XTT, allowing sensitive colorimetric assays for the determination of cell viability, was used to detect the antiviral activity of drugs. Montelukast, showing more than 80% inhibition of ZIKV infection, was selected for further study. To confirm the antiviral activity of montelukast, whose chemical structure is shown in Figure 1A, against ZIKV infection, plaque reduction assay was employed in two different cell types, BHK-21 and Vero E6. First, we tested the antiviral efficacy of montelukast against infection of ZIKV strain SZ01. It showed that montelukast inhibited ZIKV strain SZ01 infection in a dose-dependent manner with a similar IC50 value of 1.14 ± 0.19 μM in BHK-21 cells (Figure 1B and Table 2) and 1.35 ± 0.17 μM in Vero E6 cells (Figure 1E and Table 2). It is known that ZIKV is classified into two distinct phylogenetic lineages, the African and the Asian lineages; and there are intrinsic differences in the pathogenicity and virulence between these two lineages of ZIKV (Simonin et al., 2017). We then further tested if montelukast could block infection by different ZIKV strains from Asian and African lineages. As shown in Figure 1, besides ZIKV strain SZ01 (Asian lineage), montelukast also potently inhibited infection of ZIKV strain MR766 (African lineage) and FLR (Asian lineage) with IC50 values of 1.39 ± 0.13 and 1.29 ± 0.23 μM in BHK-21 cells (Figures 1C,D and Table 2) and of 1.23 ± 0.16 and 1.88 ± 0.36 μM in Vero E6 cells (Figures 1F,G and Table 2), respectively, which indicated that montelukast was effective in blocking infection of two lineages of ZIKV strains isolated from rhesus monkeys or patients in divergent regions. Furthermore, the antiviral activity of montelukast against ZIKV strain SZ01 infection in the human astrocytoma cells U-251 MG was tested. It showed that montelukast was also effective in inhibiting the infection of ZIKV in U-251 MG cells with IC50 value of 4.06 ± 0.13 μM (Table 2 and Supplementary Figure S1). The cytotoxicity of montelukast on BHK-21 cells, Vero E6 cells, and U-251 MG cells was evaluated by using CCK-8 to exclude the possibility of cytotoxicity-induced viral reduction (Figure 2 and Table 3).

**TABLE 2:** Antiviral activity of montelukast against ZIKV, DENV, and YFV in different cell lines.

Cell type	Virus	IC50 (μM)
BHK-21	ZIKV SZ01	1.14 ± 0.19
	ZIKV MR766	1.39 ± 0.13
	ZIKV FLR	1.29 ± 0.23
	DENV-2	1.03 ± 0.11
	YFV 17D	1.11 ± 0.07
Vero E6	ZIKV SZ01	1.35 ± 0.17
	ZIKV MR766	1.23 ± 0.16
	ZIKV FLR	1.88 ± 0.36
	DENV-2	0.98 ± 0.04
	YFV 17D	1.42 ± 0.09
U-251 MG	ZIKV SZ01	4.06 ± 0.13

*The 50% inhibitory concentration (IC50) of montelukast was presented as means ± SD (standard deviation). ZIKV, Zika virus; DENV, dengue virus; YFV, yellow fever virus.*

**TABLE 3:** The cytotoxicity of montelukast in different cell lines.

Cell type	CC50 (μM)
BHK-21	47.78 ± 1.83
Vero E6	46.22 ± 3.25
U-251 MG	25.05 ± 0.59

*The 50% cytotoxicity concentration (CC50) is presented as means ± SD (standard deviation).*

**FIGURE 3:**
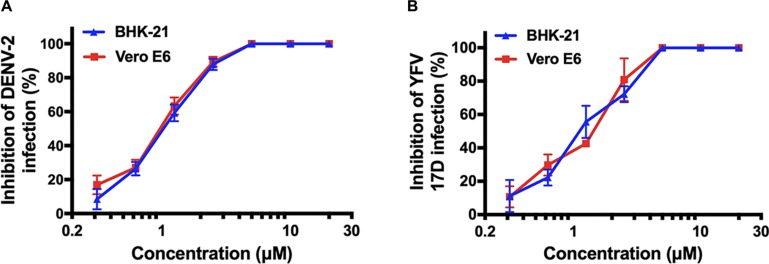
Antiviral activity of montelukast against other two flaviviruses [dengue virus (DENV)-2 and yellow fever virus (YFV) 17D] in two host cells. Dose-dependent inhibition of DENV-2 **(A)** and YFV 17D **(B)** by montelukast in BHK-21 and Vero E6 cells. All experiments were carried out in triplicate, and the error bars stand for standard deviation (SD). The 50% inhibitory concentration (IC50) is presented as means ± SD and summarized in Table 2.

Following this, we were interested in whether montelukast could inhibit infection of DENV and YFV, the other two important mosquito-borne flaviviruses circulating all around the world. It was found that montelukast could also effectively block infection of DENV-2 and YFV 17D with IC50 values of 1.03 ± 0.11 and 1.11 ± 0.07 μM in BHK-21 cells and of 0.98 ± 0.04 and 1.42 ± 0.09 μM in Vero E6 cells, respectively (Figures 3A,B and Table 2). These results suggested that montelukast may possess a broad antiviral activity against infection of a wide spectrum of flaviviruses.

### Montelukast Blocked Infection at the Early Stage of Virus Life Cycle

**FIGURE 4:**
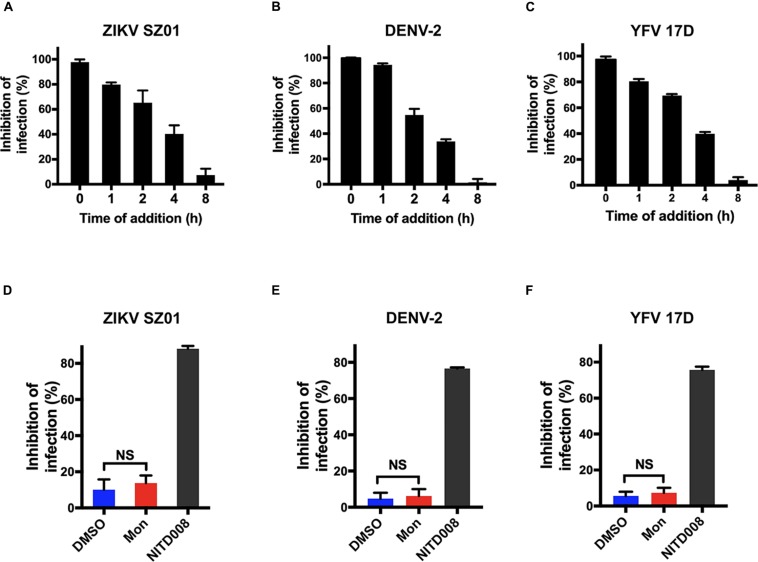
Montelukast inhibited flavivirus infection at the early stage of virus life cycle. Time of addition experiment of montelukast against Zika virus (ZIKV) SZ01 **(A)**, dengue virus (DENV)-2 **(B)**, and yellow fever virus (YFV) 17D **(C)**. Montelukast hardly inhibited ZIKV SZ01 **(D)**, DENV-2 **(E)**, and YFV 17D **(F)** infection at post-entry stage. NITD008, an adenosine analog, inhibiting flaviviruses RNA replication by terminating viral RNA synthesis was included as control. All experiments were carried out in triplicate, and the error bars stand for standard deviation (SD). The data are presented as means ± SD. NS, not significant. Student’s two-tailed *t*-test.

Montelukast is the leukotriene receptor antagonists successfully developed for treatment of asthmatic patients (Bakhireva et al., 2007; Cavero-Carbonell et al., 2017). However, little is known about its potential mechanism of the antiviral activity against ZIKV, DENV, and YFV. In order to determine at which stage of the viral life cycle the montelukast executed antiviral function, we first investigated the influence of the time of addition on plaque formation in BHK-21 cells. The montelukast was added with virus simultaneously (time 0) or at different time points after viral infection. At 16 hpi, the supernatant was discarded, and the cells were then washed to perform the plaque assay for evaluating inhibitory effects of montelukast at different time points. As shown in Figure 4A, simultaneous addition of montelukast and ZIKV to BHK-21 cells (time 0) maximally decreased the plaque numbers. With time lapsed from 1 to 4 h, the inhibitory activity of montelukast gradually decreased. The inhibitory effect on plaque formation was barely observed if montelukast was added 8 hpi, indicating that montelukast hardly suppressed flavivirus RNA replication in the late stage. It was confirmed by the assay of viral RNA replication (Figure 4D) where montelukast and NITD008, an adenosine nucleoside inhibitor targeting the stage of flavivirus RNA replication (Yin et al., 2009; Deng et al., 2016a), were both added 4 hpi. The patterns of the time of addition experiment and assay of viral RNA replication were similar for DENV-2 (Figures 4B,E) and YFV 17D (Figures 4C,F), suggesting that montelukast inhibited flavivirus infection at the early stage.

**FIGURE 5:**
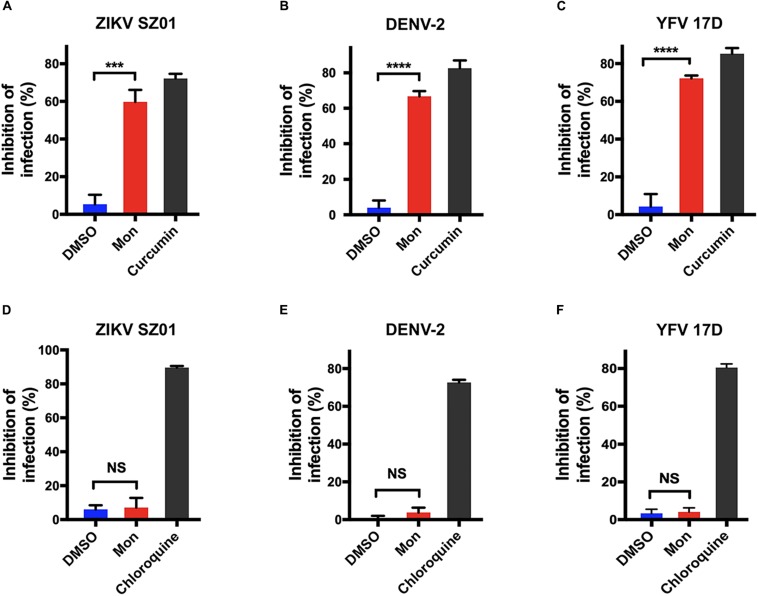
Montelukast blocked flaviviruses adsorption, not internalization. Virus adsorption assay of montelukast against Zika virus (ZIKV) SZ01 **(A)**, dengue virus (DENV)-2 **(B)**, and yellow fever virus (YFV) 17D **(C)**; and curcumin, an inhibitor known to block virus adsorption, was included as control. Virus internalization assay of montelukast against ZIKV SZ01 **(D)**, DENV-2 **(E)**, and YFV 17D **(F)**, and chloroquine, an inhibitor known to block virus internalization, was included as control. All experiments were carried out in triplicate, and the error bars stand for standard deviation (SD). The data are presented as means ± SD. NS, not significant; ***P < 0.001; ****P < 0.0001, Student’s two-tailed *t*-test.

As the early stage of virus infection consists of processes including viral attachment and internalization, the effect of montelukast on the virus adsorption or internalization was evaluated separately as previously described (Talarico et al., 2005; Talarico and Damonte, 2007) to ascertain the particular inhibitory step at the early stage of ZIKV life cycle. BHK-21 cells were incubated simultaneously with ZIKV and montelukast on ice, a low-temperature condition where the virus attachment to the cell is the only event of the virus life cycle that occurs (Talarico et al., 2005). As shown in Figure 5A, the montelukast blocked ZIKV attachment as the amount of cell-bound virus particles highly decreased in the presence of montelukast to a comparable extent caused by curcumin, a known inhibitor that suppressed ZIKV attachment to the cell (Mounce et al., 2017). The inhibitory effect of montelukast on the subsequent step, that is, virus internalization, was next analyzed. After virus adsorption on ice, the unbound virus was removed, and the montelukast was subsequently added to the cell culture. The temperature was immediately shifted to 37°C to initiate virus penetration, and plaque assay was performed thereafter. The chloroquine phosphate, a known inhibitor blocking virus internalization, was included as control. It showed that montelukast has little effect on the internalization of ZIKV (Figure 5D). The patterns of effect of montelukast on adsorption or internalization of DENV-2 (Figures 5B,E) and YFV 17D (Figures 5C,F) were similar to those of ZIKV. Overall, these results indicated that the antiviral action of montelukast mainly took place at the adsorption step in the early stage of virus life cycle.

### Montelukast Irreversibly Inhibited Viral Infectivity and Induced Release of Viral Genome RNA

Next, because montelukast obstructed flavivirus infection at the adsorption step, it became natural to question whether the inhibition action is reversible or not. Infectivity inhibition reversibility assay was then employed for clarification.

**FIGURE 6:**
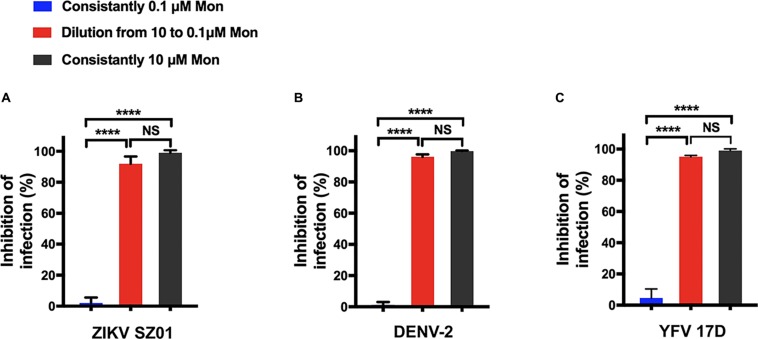
Montelukast irreversibly disrupted viral infectivity. Infectivity inhibition reversibility assay of montelukast against Zika virus (ZIKV) SZ01 **(A)**, dengue virus (DENV)-2 **(B)**, and yellow fever virus (YFV) 17D **(C)**. All experiments were carried out in triplicate, and the error bars stand for standard deviation (SD). The data are presented as means ± SD. NS, not significant; ****P < 0.0001, Student’s two-tailed *t*-test.

ZIKV was first treated with montelukast at 10 μM, a concentration sufficient to produce approximately 90% inhibition of infectivity. Then the mixture of virus and montelukast was diluted 100-fold to 0.1 μM, a concentration expected to produce negligible inhibition immediately before plaque assay was performed. ZIKV constantly treated with 10 μM or constantly treated with 0.1 μM of montelukast was also included for plaque assay. As shown in Figure 6A, dilution of montelukast could not abolish the inhibitory effect, indicating that the inhibition of ZIKV infectivity is irreversible. Similarly, no reversibility of inhibition was observed for DENV-2 (Figure 6B) and YFV 17D (Figure 6C).

**FIGURE 7:**
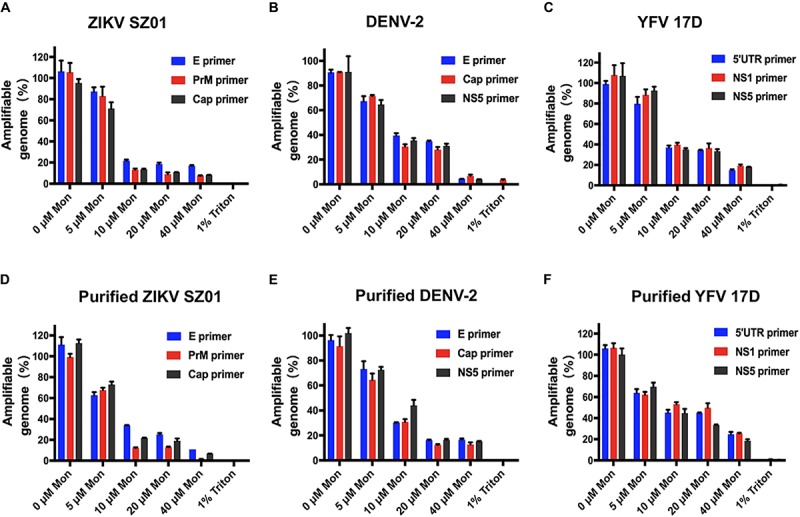
Degradation of released genomic RNA of flaviviruses mediated by montelukast treatment in an RNase digestion assay. The release and degradation of genomic RNA of Zika virus (ZIKV) SZ01 **(A)**, dengue virus (DENV)-2 **(B)**, yellow fever virus (YFV) 17D **(C)**, and purified virions of ZIKV SZ01 **(D)**, DENV-2 **(E)**, and YFV 17D **(F)** were detected by using their respective primers targeting different regions in the viral genome. All experiments were carried out in triplicate, and the error bars stand for standard deviation (SD). The data are presented as means ± SD.

The irreversibility of inhibitory effect of montelukast aroused our interest in whether the release of viral genome RNA is induced by montelukast. Then, the potential viral RNA release was measured by an RNase digestion assay as described previously (Lok et al., 2012; Yu et al., 2017). The RNA genomes of intact virions would be protected from RNase digestion, whereas the virions whose integrity was disrupted by treatment would be susceptible to RNA degradation by RNase digestion. As shown in Figure 7A, the viral genomic RNA of ZIKV treated with montelukast was digested by micrococcal nuclease in a dose-dependent manner, and almost 80% genomic RNA of ZIKV particles treated with 10 μM of montelukast was digested. Montelukast treatment caused similar RNA genome degradation upon RNase digestion for DENV-2 (Figure 7B), for YFV 17D (Figure 7C), and for purified virions of ZIKV, DENV-2, and YFV 17D (Figures 7D–F), which suggested that montelukast could disrupt the integrity of flaviviruses and hence irreversibly destroy the infectivity of flaviviruses.

### Montelukast Blocked Vertical Transmission of Zika Virus in Pregnant Mice

**FIGURE 8:**
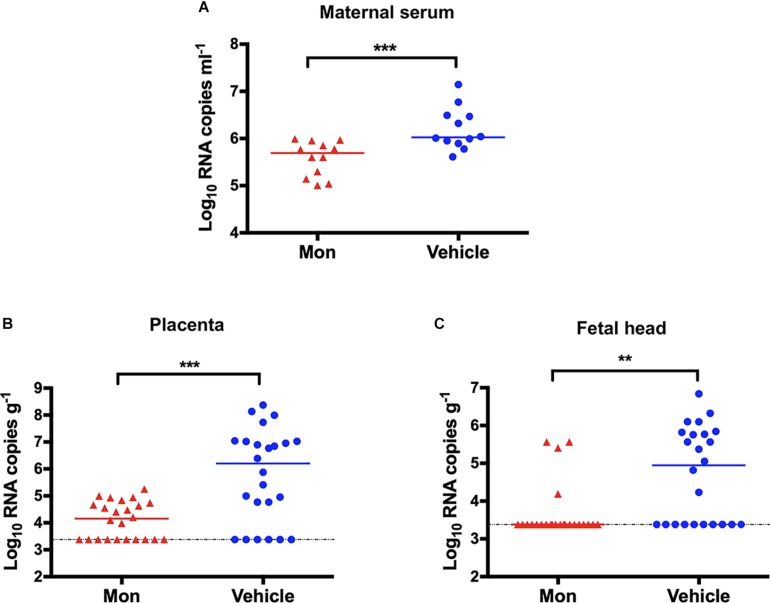
Protection against vertical transmission of Zika virus (ZIKV) in montelukast-treated pregnant C57BL/6 mice. **(A)** Viremia of pregnant C57BL/6 mice (*n* = 12 in each group). **(B)** Viral RNA loads in the placentas (*n* = 24 in each group). Two embryos of each pregnant mouse were randomly collected, and the viral RNA load in each placenta was determined. **(C)** Viral RNA loads in the fetal heads (*n* = 24 in each group). The viral RNA load in the fetal head of each collected embryo was determined. The bars reflect median values. The horizontal dotted lines represent limits of detection. **P < 0.01; ***P < 0.001, Mann–Whitney test.

To evaluate whether montelukast could block the vertical transmission of ZIKV, pregnant C57BL/6 mice were infected with ZIKV as described previously (Wu et al., 2016; Yu et al., 2017) and were then treated with montelukast or vehicle control. As shown in Figure 8A, treatment with montelukast could reduce viremia in the pregnant C57BL/6 mice infected with ZIKV (*P* = 0.0006). Meanwhile, the viral RNA loads of placentas from pregnant mice treated with montelukast were greatly reduced compared with those from vehicle-treated mice (*P* = 0.0005), and the infection rate declined from 18/24 to 14/24 (Figure 8B). And montelukast treatment led to significant decrease both of viral RNA load in fetal head (*P* = 0.0014, Figure 8C) and of infection rate from 14/24 to 4/24. These results suggested that montelukast may lower the infection rate of the fetuses by inactivating ZIKV virions either before or after the virus invaded from the placenta to fetus, hence protecting against vertical transmission of ZIKV in pregnant mice, just like Z2, a peptide-based viral inactivator (Yu et al., 2017).

### Montelukast Protected A129 Mice From Lethal Challenge With Zika Virus

**FIGURE 9:**
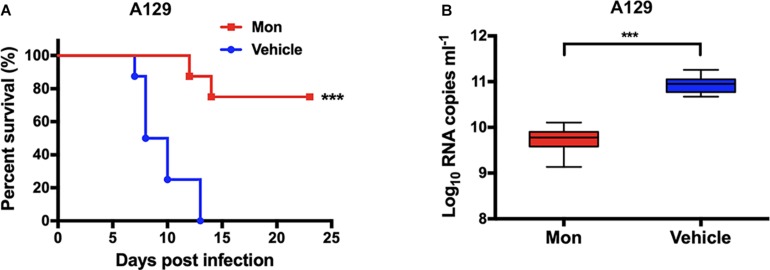
Protective activity of montelukast against lethal Zika virus (ZIKV) infection in type I interferon receptor-deficient A129 mice. **(A)** Survival of ZIKV-infected A129 mice. ***P < 0.001, log-rank (Mantel–Cox) test. **(B)** Viral RNA loads in sera of ZIKV-infected A129 mice. Whiskers: 5th–95th percentile. ***P < 0.001, Mann–Whitney test.

Finally, the antiviral efficacy of montelukast against ZIKV infection was evaluated in the recently established A129 (interferon alpha/beta receptor-deficient) (Dai et al., 2016; Shan et al., 2017; Yu et al., 2017; Tai et al., 2019) mouse model. The A129 mice were challenged with ZIKV i.p. and then were subsequently treated with montelukast or vehicle. The mice treated with vehicle exhibited a 100% mortality rate at 13 dpi as shown in Figure 9A. Instead, 75% of the A129 mice treated with montelukast were protected from death caused by ZIKV infection (*P* = 0.0003, log-rank test). The viral loads in A129 mice treated with montelukast at 2 dpi were much lower than those of mice treated with vehicle (*P* = 0.0002, Figure 9B). Although montelukast suppressed ZIKV infection at the early stage of viral life cycle, consecutive injections after ZIKV invading into cells could still render some protection possibly by irreversibly disrupting newly produced viruses and hence preventing infection of more target cells.

## Discussion

ZIKV is one of the mosquito-borne flaviviruses discovered about 70 years ago and has spread all over the world in recent years, which causes Guillain–Barré syndrome, congenital Zika syndrome, and damage to testicular tissue (Brasil et al., 2016; Baud et al., 2017; Gurung et al., 2019; Peixoto et al., 2019). Currently, there are no vaccines or drugs approved for prevention and treatment of ZIKV infection, leading to the necessity and urgency of the development of anti-ZIKV therapy. Repurposing screen of clinically approved drugs is a practical way to deal with the outbreak of emerging and reemerging infectious disease threats such as Middle East respiratory syndrome (MERS) and Ebola (Johansen et al., 2013; de Wilde et al., 2014; Dyall et al., 2014; Kouznetsova et al., 2014; Wang et al., 2017). By employing this method, some drugs have been found to be effective against ZIKV infection (Xu et al., 2016). We also identified a marketed anti-asthmatic drug, montelukast, which has not been reported as far as we know.

Montelukast, one of the leukotriene receptor antagonists, is the most prescribed cysteinyl leukotriene 1 (CysLT1) antagonist safely used in asthmatic patients for many years (Hoxha et al., 2017). It is interesting that montelukast exhibited potential antiviral efficacy against ZIKV *in vitro* and *in vivo*. Montelukast not only inhibited infections of several types of flaviviruses, including ZIKV of Asian and African lineages, DENV-2, and YFV 17D, in two kinds of cells, but also protected A129 mice from lethal ZIKV challenge and blocked vertical transmission of ZIKV in pregnant mice. The inhibition of ZIKV infectivity by montelukast is irreversible probably owing to the disruption of the integrity of ZIKV virions and the release of ZIKV genomic RNA. It should be noted that the concentration needed to cause 50% degradation of the genome was a little bit higher than the concentration required to yield 50% reduction in infectivity, which was also reported in the studies from others (Lok et al., 2012; Yu et al., 2017). Just as mentioned in their study, this might be caused by the use of more viruses in the genome degradation assay or by some virions having only partial genomes released to be still protected from degradation and to be likely non-infectious. Interestingly, montelukast has no effect on the genomic RNA release of EV71, an unenveloped RNA virus (Supplementary Figure S3A, and Supplementary Figures S3B–E show the amplicons of ZIKV SZ01, DENV-2, YFV 17D and EV71 that were resolved by agarose gel electrophoresis). Besides, montelukast also exhibited an antiviral activity against VSV-GFP (Supplementary Figure S2), which is an enveloped non-flavivirus, suggesting that the montelukast probably targeted to the lipid membrane of the viral shell, not to the envelope protein of the virus. On the other hand, the proven pharmacological action of montelukast could favor its antiviral effect as concomitant effects. Leukotrienes are important in mediating the vascular leakage caused by DENV infection-induced mast cell activation, leading to increased vascular permeability, which may result in hemorrhage within internal organs and leakage of plasma into the tissues, the distinctive features of dengue shock syndrome (DSS) and dengue hemorrhagic fever (DHF) (Ahmad et al., 2018). Montelukast, the leukotriene receptor antagonist, could reduce the vascular permeability and restore the vascular integrity to prevent vascular leakage and hemorrhaging (St John, 2013; St John et al., 2013). Hence, it is possible that the modulation of inflammation by montelukast would contribute to the *in vivo* anti-ZIKV effects. Certainly, other putative mechanisms underlying the antiviral action of montelukast, such as the blockage of binding of ZIKV E protein to its cell receptor, cannot be totally excluded. However, the receptor utilized by ZIKV is still controversial (Govero et al., 2016; Nowakowski et al., 2016; Retallack et al., 2016; Chen et al., 2018); therefore, more investigations will be needed in the following research.

The major sequela caused by congenital ZIKV infection is microcephaly, which is evidenced in humans and animal models (Tang et al., 2016; Cui et al., 2017), and pregnant women are the direct victims of the ZIKV infection of fetus, making it vitally important that the treatment for pregnant women infection by ZIKV should be very cautious. Luckily, montelukast is classified as category B in the FDA pregnancy category and has been successfully used for treatment of pregnant women with asthma (Bakhireva et al., 2007; Cavero-Carbonell et al., 2017). Animal studies showed no adverse effects on embryo and fetal development at oral doses up to 400 mg/kg/day in rats or up to 100 mg/kg/day in rabbits, as described in the product information of montelukast (trade name Singulair^®^) produced by Merck Sharp & Dohme, Ltd. With the combination of the fact that montelukast blocked vertical transmission of ZIKV in pregnant mice and its safety profile for pregnant women, it is promising that montelukast could be used for treatment of pregnant women infected with ZIKV. And more surprising and exciting to us is that montelukast displayed neuro-protective activity in several animal models. Montelukast may improve the fiber connectivity and long-term functional recovery after brain ischemia caused by stroke, enhancing recruitment and maturation of oligodendrocyte precursor cells (Gelosa et al., 2019), and may ameliorate amyloid-β-induced memory impairment via inhibition of neuro-inflammation and apoptosis in mice (Lai et al., 2014). Moreover, treatment of old animals with montelukast reduces neuro-inflammation, elevates hippocampal neurogenesis, and improves learning and memory (Marschallinger et al., 2015). The montelukast-mediated restoration of cognitive function correlates with the increased neurogenesis. In consideration of the role of neuro-inflammation played in the occurrence and development of microcephaly induced by ZIKV infection, it is hoped that montelukast may be used for relief from neuro-inflammation.

Taken together, montelukast exhibited potent antiviral efficacy against several flaviviruses, including ZIKV of Asian and African lineages, DENV-2, and YFV 17D via the irreversible inhibition of infectivity caused by disruption of the integrity of virions, conferred protection from lethal ZIKV challenge and blocked the vertical transmission of ZIKV, indicating its potential application for treatment of ZIKV infections, especially in high-risk populations such as pregnant women considering its safety profile and neuro-protective activity.

## Data Availability Statement

All datasets generatedfor this study are included in the article/Supplementary Material.

## Ethics Statement

The animal studywas reviewed and approved by the Shanghai Public Health Clinical Center Laboratory Animal Welfare & Ethics Committee.

## Author Contributions

PZ conceived and designed the experiments. YC, YL, and XW performed the experiments. YC and YL analyzed the data. YC, YL, and PZ wrote the manuscript.

## Conflict of Interest

The authors declare that the research was conducted in the absence of any commercial or financial relationships that could be construed as a potential conflict of interest. The reviewer C-FQ declared a past co-authorship with one of the authors PZ to the handling Editor.
