# Supplementary material for: Montelukast, an Anti-asthmatic Drug, Inhibits Zika Virus Infection by Disrupting Viral Integrity
Source: Front Microbiol. 2020 Jan 30;10:3079. doi: 10.3389/fmicb.2019.03079 (PMC7002393; doi:10.3389/fmicb.2019.03079)
Supplement: Supplementary file 1 [file Data_Sheet_1.PDF]

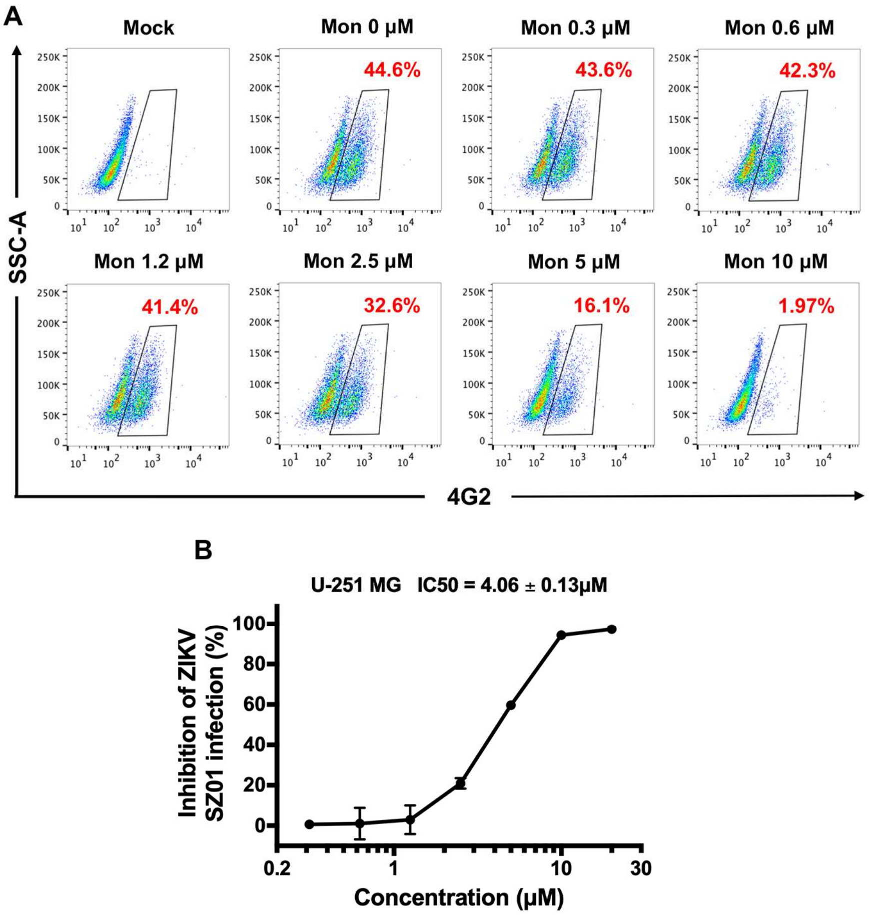

FIGURE S1 | Montelukast exhibited anti-ZIKV activity in U-251 MG cells. The human astrocytoma U-251 MG cells were infected with ZIKV strain SZ01 at MOI of 1 after viruses incubating with the indicated concentration of montelukast for 1h. The cells were harvested and analyzed by flow cytometry at 40 hpi. **(A)** Red numbers in the representative dot plots indicated the percentage of ZIKV-infected U-251 MG cells. SSC-A stands for Side Scatter - Area. **(B)** Dose response curve of montelukast inhibiting ZIKV infection. All experiments were carried out in triplicate and the error bars stand for standard deviation (SD). The 50% inhibitory concentration (IC50) was presented as means ± SD and summarized in TABLE 1.

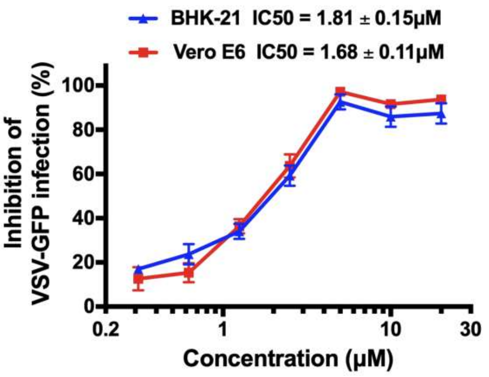

FIGURE S2 | Antiviral activity of montelukast against enveloped none-flavivirus (VSV-GFP) in two host cells. Dose-dependent inhibition of VSV-GFP by montelukast in BHK-21 and Vero E6 cells. All experiments were carried out in triplicate and the error bars stand for standard deviation (SD). The 50% inhibitory concentration (IC50) was presented as means ± SD.

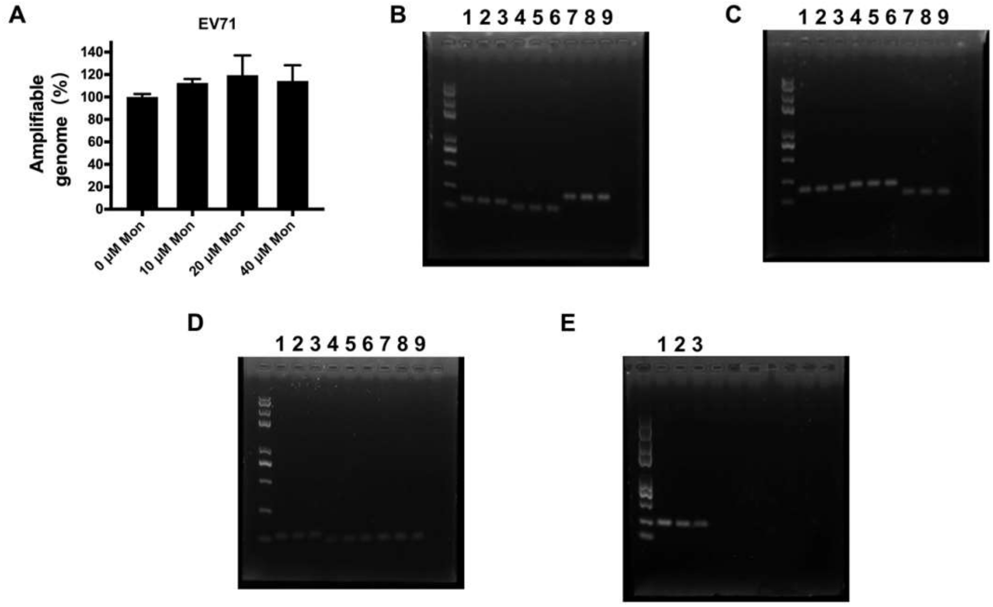

FIGURE S3 | RNase digestion assay of EV71 mediated by montelukast treatment **(A)** and the amplicons of ZIKV SZ01 **(B)**, DENV-2 **(C)**, YFV 17D **(D)** and EV71 **(E)** resolved by agarose gel electrophoresis. **(A)** RNase digestion assay of EV71 was carried out in triplicate and the error bars stand for standard deviation (SD). **(B)** The amplicons of ZIKV SZ01 by primers pair Cap F1 and Cap R1 (lane 1-3), E F1 and E R1 (lane 4-6) and PrM F1 and PrM R1 (lane 7-9). **(C)** The amplicons of DENV-2 by primers pair 5-1F and 5-1R (lane 1-3), 5M-1F and 5M-1R (lane 4-6) and 3M-2F and 3M-2R (lane 7-9). **(D)** The amplicons of YFV 17D by primers pair F3 and R3 (lane 1-3), YFVdual-fwd-vac and YFVdual-rv-vac (lane 4-6) and F and R (lane 7-9). **(E)** The amplicons of EV71 by primers pair EV71-F and EV71-R (lane 1-3). The DNA ladders are 8000 bp, 5000 bp, 3000 bp, 2000 bp, 1000 bp, 750 bp, 500 bp, 250 bp and 100 bp from top to bottom.
